# Association between *5-HT2A*, *TPH1 *and *GNB3 *genotypes and response to typical neuroleptics: a serotonergic approach

**DOI:** 10.1186/1471-244X-7-22

**Published:** 2007-05-23

**Authors:** Sami Anttila, Olli Kampman, Ari Illi, Riikka Rontu, Terho Lehtimäki, Esa Leinonen

**Affiliations:** 1 University of Tampere, Medical School, 33014 University of Tampere, Finland; 2 Laboratory of Atherosclerosis Genetics, Department of Clinical Chemistry, Centre for Laboratory Medicine, Tampere University Hospital, Teiskontie 35, PL 2000, 33521 Tampere, Finland; 3 Seinäjoki Hospital District, Department of Psychiatry, Hanneksenrinne 7, 60220 Seinäjoki, Finland; 4 Kanta-Häme Central Hospital, Department of Psychiatry, 13530 Hämeenlinna, Finland; 5 Tampere University Hospital, Department of Psychiatry, 33380 Pitkäniemi, Finland

## Abstract

**Background:**

Schizophrenia is a common psychiatric disease affecting about 1% of population. One major problem in the treatment is finding the right the drug for the right patients. However, pharmacogenetic results in psychiatry can seldom be replicated.

**Methods:**

We selected three candidate genes associated with serotonergic neurotransmission for the study: serotonin 2A (*5-HT2A*) receptor gene, tryptophan hydroxylase 1 (*TPH1*) gene, and G-protein beta-3 subunit (*GNB3*) gene. We recruited 94 schizophrenia patients representing extremes in treatment response to typical neuroleptics: 43 were good responders and 51 were poor responders. The control group consisted of 392 healthy blood donors.

**Results:**

We do, in part, replicate the association between *5-HT2A *T102C polymorphism and response to typical neuroleptics. In female patients, C/C genotype was significantly more common in non-responders than in responders [OR = 6.04 (95% Cl 1.67–21.93), p = 0.005] or in the control population [OR = 4.16 (95% CI 1.46–11.84), p = 0.005]. *TPH1 *A779C C/A genotype was inversely associated with good treatment response when compared with non-responders [OR = 0.59 (95% Cl 0.36–0.98), p = 0.030] or with the controls [OR = 0.44 (95% CI 0.23–0.86, p = 0.016], and *GNB3 *C825T C/T genotype showed a trend-like positive association among the male patients with a good response compared with non-responders [OR = 3.48 (95% Cl 0.92–13.25), p = 0.061], and a clearer association when compared with the controls [OR = 4.95 (95% CI 1.56–15.70), p = 0.004].

**Conclusion:**

More findings on the consequences of functional polymorphisms for the role of serotonin in the development of brain and serotonergic neurotransmission are needed before more detailed hypotheses regarding susceptibility and outcome in schizophrenia can be formulated. The present results may highlight some of the biological mechanisms in different courses of schizophrenia between men and women.

## Background

Schizophrenia is a devastating disease which affects about one percent of people world-wide [1]. Recent studies suggest that the interaction between brain dopaminergic and serotonergic systems is of major significance in the neuropathology and treatment of schizophrenia [2].

The pharmacogenetics of schizophrenia has focused on studying dopamine and serotonin related genes [3]. Several studies have found association between serotonergic genes and response to clozapine [3]. In typical antipsychotics, only few studies were related to dopamine receptor genes and most results were negative for response prediction [3,4]. Several lines of evidence indicate an important role of serotonergic modulation in the mechanism of action of typical neuroleptics. Although typical neuroleptics act via dopamine-D2 receptor blockade, they all (including haloperidol) affect both dopaminergic and serotonergic receptors [5]. Altered serotonergic response in brain have been reported in treatment-refractory patients with schizophrenia [6]. Moreover, haloperidol has been reported to increase 5-HT release in prefrontal cortex [7]. Systemic administration of apomorphine, a dopamine receptor agonist, has been found to both decrease and increase striatal 5-HT release and increase 5-HT release in the hippocampus [7]. Serotonin 5-HT2C receptor affinity is also negatively correlated with the dose of typical neuroleptics. Thus, increasing serotonin 5-HT2C receptor antagonist affinity lowers the potency of typical neuroleptics [8]. It is possible that actions of serotonin receptors in addition to dopamine D2 receptors are relevant to the actions of typical neuroleptics [9].

One study suggests that serotonin 2A (*5-HT2A*) receptor gene polymorphism is associated with treatment response to conventional antipsychotics [10]. In meta-analyses *5-HT2A *T102C polymorphism has been associated with the risk of schizophrenia [11,12]. The *5-HT2A *T102C C-allele specific methylation has been associated with increased 5-HT2A receptor expression in human temporal cortex [13], and allele-specific methylation has been proposed as a modulator in gene-environment interactions [14].

So far there have been no pharmacogenetic studies of tryptophan hydroxylase 1 (*TPH1*) gene polymorphism in schizophrenia. Genetic variation of *TPH1 *gene has been associated with the risk of schizophrenia in two recently published studies [15,16]. The *TPH *779 A-allele has been connected with lower CSF 5-HIAA concentrations in healthy men [17]. TPH1 is mainly expressed in the pineal gland, and it is responsible for peripheral serotonin response, in contrast to TPH2, which is expressed in the raphe nucleus and directly affects the serotonergic pathways of the brain [18]. The importance of TPH1 comes through possible biological susceptibility through abnormal serotonin levels during the development of brain [19], or through abnormal response to stress [20], which may further indicate mental disorders of several types, and particularly schizophrenia.

The G-protein beta-3 subunit (*GNB3*) C825T polymorphism (T-allele) results in increased intracellular signal transduction in G-protein coupled receptors [21]. TT genotype or T allele carriers of C825T polymorphism have been associated in three studies with treatment response to serotonin selective reuptake inhibitors in the treatment of depression [22-24]. *GNB3 *C825T polymorphism has been associated with treatment response in various antipsychotic drugs [25] as well as clozapine induced weight gain [26]. In addition, GNB3 polymorphism may predict response of several other serotonergic treatments: triptans [27], sibutramine [28], and electroconvulsive treatment [29]. It is thus suggested that G-protein beta-3 subunit may mediate the serotonergic effect at the second messenger cascade level [30].

We hypothesized that these functional SNPs would alter serotonergic neurotransmission and stress response in the CNS, and might thus be suitable candidate genes for a pharmacogenetic study with typical neuroleptics. The hypothesis was tested in a study design including two groups of schizophrenia patients with a well-documented response, either good or poor to typical neuroleptics, and resembling two different endophenotypes in their course of the disorder and clinical response to treatment. As *TPH1 *polymorphism is particularly interesting in relation to stress response, which is in part regulated by estrogen [31], we deemed it appropriate to analyse differences in genotype distributions between the men and women in our sample. The genotype distributions were compared between the schizophrenia subgroups and the distributions of control group were also used as a reference.

## Methods

### Patients and controls

The patients were 94 unrelated Finnish patients with schizophrenia. All patients met the criteria for schizophrenia of the Diagnostic and Statistical Manual of Mental Disorders (DSM)-IV. Group 1 (responders) consisted of patients with schizophrenia who had experienced sufficient and sustained response to treatment with typical antipsychotics. The patients in group 2 (non-responders) had failed to respond to treatment with at least two different typical antipsychotics. Because of poor response to conventional neuroleptics, clozapine was initiated in all patients in the non-responders' group. The control sample consisted of 392 healthy blood donors (Table 1).

**Table 1:** Characteristics of patient and control groups.

	Sex M/F	Age years	Age at onset years
Responders (n = 43)	17/26	48.1 ± 11.4	32.2 ± 9.5
Non-responders (n = 51)	29/22	44.1 ± 10.5	27.5 ± 10.2^1^
Controls	212/180	44.4 ± 11.1	

^1 ^p = 0.02 compared to responders (chi-square test)

The study was performed in compliance with the code of ethics of the World Medical Association (Declaration of Helsinki) and the standards established by the local ethics committee. The participants gave written informed consent.

The responder group (group I, n = 43; 17 men, 26 women) consisted of patients who had experienced a sufficient and long-lasting response to treatment with conventional antipsychotics. Assessment of response was based on information in hospital and mental health care records and a personal interview with each patient. Before initiation of antipsychotic treatment, the severity of schizophrenia symptoms had to be ≥ 4 according to the Clinical Global Impression Scale (CGI). The patient records were thoroughly reviewed from the time of the index hospitalization or acute treatment in mental health care to ensure that both the patient's psychotic symptoms had disappeared and the level of functioning had returned to a level corresponding to that prior the psychotic episode. In addition, a clinical interview was conducted with each patient during the time of the study to ascertain that the good response had been sustained. The main conventional antipsychotics used in the index treatment period included chlorpromazine in fifteen, perphenazine, in nine, chlorprothixene in six, zuclopentixole in five, thioridazine in four, haloperidol in three and flupentixole in one of the cases.

The non-responders' group (group II, n = 51; 29 men, 22 women) comprised such patients on clozapine medication who had failed to respond to treatment with two different conventional antipsychotics on at least two different occasions in a hospital setting. In each index treatment period with conventional antipsychotics the lowest accepted daily dose was 400 mg chlorpromazine equivalent for a minimum of four weeks. Prior to the initiation of clozapine treatment the severity of symptoms of schizophrenia had to be ≥ 4 on the CGI Scale, and at least one of the following symptoms had to be present: conceptual disorganization, suspiciousness, hallucinatory behavior, or unusual thought content. The main conventional antipsychotics used in the two index treatment periods included chlorpromazine in 23/8, perphenazine, in 5/2, chlorprothixene in 6/9, zuclopentixole in 6/13, thioridazine in 5/2, haloperidol in 4/13, levomepromazine in 2/2, flupentixole in 0/1 and sulpiride in 0/1 of the cases (the numbers indicating first treatment period/second treatment period).

### DNA extraction and genotyping of the *TPH1 *A218C, A779C, *GNB3 *C825T and *5-HT2A *T102C polymorphisms

Genomic DNA was extracted from peripheral blood leukocytes using a commercially available kit and BioRobot M48 Workstation according to manufacturer's instructions (Qiagen Inc., Hilden, Germany). DNA samples were genotyped by employing the 5' nuclease assay and fluorogenic allele-specific TaqMan MGB probes [32] using the ABI Prism 7900 HT Sequence Detection System (Applied Biosystems, Foster City, CA, USA). The nucleotide sequences of primers and probes used in the PCR were deduced from published sequences deposited in the GenBank and Celera databases and synthesized in conjugation with Applied Biosystems. Validated genotyping assays were used for the TPH1 A779C (rs1799913), 5-HT2A T102C (rs6313) and GNB3 C825T (rs6489738) polymorphisms and custom assay for the TPH1 A218C (rs1800532). PCR reaction containing genomic DNA, 1 × Universal PCR Master Mix, 900 nM of each primer and 200 nM of each probe was performed in 96-well plates using the standard protocol in a total volume of 25 μl. End-point fluorescence was measured and genotype calling carried out by the allelic discrimination analysis module after PCR resulting in clear identification of three genotypes for all polymorphisms studied. All genotyping was performed blind to the clinical formation.

Genotyping of 5-HT2A T102C polymorphism failed in one patient (a non-responder) and in six control subjects.

### Statistical analysis

We analysed the distributions of genotypes in both the responders' and the non-responders' group in comparison with the control group, and between both patient groups. The differences of genotype distributions were calculated using Pearson Chi-Square test and odds ratios between groups were calculated from 2 × 2 contingency tables. Differences in age, age at onset and antipsychotic dose between genotype groups were calculated with ANOVA, but no significant differences were found (p > 0.05).

In determining statistical power with the analyses of allele frequencies between schizophrenia subgroups, we assumed 80% of the patients in each group to be correctly classified. With this assumption, the level of statistical power with this analysis was ~0.9. For the analyses in comparisons between patient subgroups and the control group, the odds ratios needed for detecting differences in genotype distributions were calculated with the power level of 0.8. The distributions of different genotypes in the control sample were used as references in these analyses. With a power level of 0.8, the threshold odds ratios between groups in each genotype were 2.5–3.1 for the *5-HT2A *polymorphism, 2.5–2.6 for the *TPH1 *polymorphism, and 2.4–3.4 for the *GNB3-825 *polymorphism. Data analysis was carried out using SPSS/Win (versions 12.0 and 14.0, SPSS Inc., Chicago, IL) and PS (version 2.1.31, (Dupont and Plummer 2004)) software. The level of statistical significance was set at 0.05. The allele frequencies of the genes studied were in Hardy-Weinberg equilibrium.

## Results

### *5-HT2A *T102C polymorphism

In female patients, the C/C genotype was more common in non-responders than in responders [OR = 6.04 (95% Cl 1.67–21.93), p = 0.005] or in the control population [OR = 4.16 (95% CI 1.46–11.84), p = 0.005] (Table 2).

**Table 2:** Distribution of *5-HT2A*, *TPH1 *and *GNB3 *gene polymorphisms in the study population.

		*5-HT2A*			*TPH1*			*GNB3*		
		
		CC	CT	TT	CC	CA	AA	CC	CT	TT
Patients	responders (n = 43)	17 (39.5)	23 (53.5)	3 (7.0)	14 (32.6)	14 (32.6)^2^	15 (34.8)	18 (41.9)	20 (46.5)	5 (11.6)
	females	9 (34.6)	14 (53.8)	3 (11.5)	8 (30.8)	9 (34.6)	9 (34.6)	14 (53.9)	7 (26.9)	5 (19.2)
	males	8 (47.1)	9 (52.9)	0 (0.0)	6 (35.3)	5 (29.4)	6 (35.3)	4 (23.5)	13 (76.5)^3^	0 (0.0)
Patients	non-responders (n = 51)	27 (54.0)	19 (38.0)	4 (8.0)	12 (23.5)	28 (54.9)	11 (21.6)	27 (53.0)	22 (43.1)	2 (3.9)
	females	16 (76.2)^1^	4 (19.0)	1 (4.8)	3 (13.6)	13 (59.1)	6 (27.3)	13 (59.1)	8 (36.4)	1 (4.5)
	males	11 (37.9)	15 (51.7)	3 (10.3)	9 (31.0)	15 (51.7)	5 (17.3)	14 (48.3)	14 (48.3)	1 (3.4)
Controls	(n = 392)	166 (43.0)	176 (45.6)	44 (11.4)	106 (27.0)	205 (52.3)	81 (20.7)	218 (55.6)	144 (36.7)	30 (7.7)
	females	77 (43.5)	81 (45.8)	19 (10.7)	42 (23.3)	90 (50.0)	48 (26.7)	107 (59.5)	60 (33.3)	13 (7.2)
	males	89 (42.6)	95 (45.4)	25 (12.0)	64 (30.2)	115 (54.2)	33 (15.6)	111 (52.4)	84 (39.6)	17 (8.0)

Statistics: ^1 ^p = 0.005, OR = 6.04 (95% Cl 1.67–21.93) compared to responders; p = 0.005, OR = 4.16 (95% CI 1.46–11.84) compared to controls

^2 ^p = 0.030, OR = 0.59 (95% Cl 0.36–0.98) compared to non-responders; p = 0.016, OR = 0.44 (95% CI 0.23–0.86) compared to controls

^3 ^p = 0.061, OR = 3.48 (95% Cl 0.92–13.25) compared to non-responders; p = 0.004, OR = 4.95 (95% CI 1.56–15.70) compared to controls

Abbreviations: *5-HT2A*: serotonin 2A receptor gene; *TPH1*: tryptophan hydroxylase 1 gene, *GNB3*: G-protein beta-3 subunit gene.

### *TPH1 *A779C polymorphism

In the total sample, *TPH1 *C/A genotype was less common in the responders' group compared with non-responders [OR = 0.59 (95% Cl 0.36–0.98), p = 0.030] or with the controls [OR = 0.44 (95% CI 0.23–0.86, p = 0.016] (Table 2).

### *GNB3 *C825T polymorphism

In males, there was a trend-like association for C/T genotype to be more common in responders than in non-responders [OR = 3.48 (95% Cl 0.92–13.25), p = 0.061]. In males, C/T genotype was more common in responders than in controls [OR = 4.95 (95% CI 1.56–15.70), p = 0.004] (Table 2).

## Discussion

The study models two endophenotypes of schizophrenia in relation to the response with conventional neuroleptics in comparison with the control subjects. The different allele distributions in patient subgroups compared with controls may indicate some part of the genetic susceptibility to schizophrenia, which has been linked to abnormal stress response, and even more importantly different subgroups of the schizophrenic syndrome. The main problem with the study methodology includes the small patient sample, especially in comparisons between genders, but on the other hand the selection criteria for both subgroups are restricted, resulting in marked differences in the clinical picture between the patient groups, and less variation in the course of the disease within the groups. The selection criteria for these subgroups resulted in more early-onset patients in the non-responder group, as could be expected [33]. There was also a trend for more female patients being selected in the responder group, but this difference was not significant. Different distributions between subgroups could in part be responsible for different treatment effects, although the subgroups were selected according to the response criteria. The comparisons between schizophrenia subgroups and control group were performed in spite of small numbers of patients, and acknowledging that the differences in allele distributions deviated only slightly between the general schizophrenia population and healthy controls, for example with the *5-HT2A *T102C polymorphism [11,12]. These comparisons were necessary due to the strict selection criteria for schizophrenia subgroups, and the distributions of the control group were used as references in these analyses.

### *5-HT2A *T102C polymorphism

We report an association between *5-HT2A *T102C polymorphism and response to typical neuroleptics, where C/C genotype was associated with poor response in female patients. Earlier, Joober et al. (1999) reported a similar association in male patients but not in female patients. The study design was very similar in both studies. However, an obvious reason for these different results may be the different gender distribution in these studies. In the study by Joober et al. there were respectively 74.4% and 71.1% males in non-responders and responders. In the present sample, there were 56.9% and 39.5% males in these categories. One explanation for the gender differences detected may be that treatment-resistant women with schizophrenia constitute a severely ill subgroup within schizophrenia [34].

In meta-analysis, *5-HT2A *T102C polymorphism has been associated with a small schizophrenia increasing effect [11,12]. Because of the small sample size in the schizophrenia population, one would predict that such a small contribution could not have been detected in the present sample, and these analyses were therefore excluded from this study.

The occurrence of *5-HT2A *T102C C-allele has been found to increase 5-HT2 receptor expression in human temporal cortex [13]. There is evidence that the density of the 5-HT2 receptors is decreased in the prefrontal cortex of patients with schizophrenia, but the results are still contradictory. This phenomenon could reflect the heterogeneity of the disease [35].

### *TPH1 *A779C polymorphism

To our knowledge, this is the first report of an association between *TPH1 *A779C polymorphism and response to typical neuroleptics. We cannot present any conclusive reason why the treatment response was inversely associated with a heterozygous genotype. However, heterosis is a common feature which may occur in up to 50% of all gene associations [36]. So far, the heterozygous C/A genotype has been reported to have a connection with higher degrees of nicotine dependence, a feature strongly associated with schizophrenia [37]. The *TPH1 *A779C A-genotype has been associated with lowered serotonin metabolite 5-HIAA levels in the CSF [17], and on the other hand the functions of TPH1 are related to stress response, which may be associated with susceptibility to schizophrenia [20]. The role of TPH1 may also be important during maturation of the serotonin neurons in the brain [19].

### *GNB3 *C825T polymorphism

Male heterozygous genotype carriers showed a better response to typical neuroleptics. So far heterosis has been reported by only one study, in which Korean male medical students were the more prone to seasonal variation the more often they carried heterozygous genotype of *GNB3 *polymorphism [38]. It should be noted that several examples suggest that heterosis is gender specific [36]. However, no association between antipsychotic medication response and the occurrence of T-allele, which has been reported to have a GNB3 expression increasing effect [21] was found in this study.

## Conclusion

Serotonergic mechanisms may have a greater impact on medication response of schizophrenia than has so far been acknowledged. This approach needs more studies including different endophenotypes of schizophrenia regarding age at onset and medication response. These subgroups are likely to have different susceptibility and treatment response altering factors. The results of this study may give rise to further hypotheses regarding the role of gender in the course and outcome of schizophrenia.

## Abbreviations

*5-HT2A*: serotonin 2A receptor gene

*TPH1*: tryptophan hydroxylase 1 gene

*GNB3*: G-protein beta-3 subunit gene

## Competing interests

The author(s) declare that they have no competing interests.

## Authors' contributions

SA: data management and statistical analyses, collecting the clinical material, writing the first draft of the manuscript

OK: data management and statistical analyses, collecting the clinical material, writing the first draft of the manuscript (authors SA and OK have equal contribution)

AI: collection of clinical material

RR: planning and performing the genotypings

TL: planning and organising the blood collection, DNA isolation and genotyping

EL: statistical analyses, coordinating the clinical study

In addition, all authors participated in composing and editing the article as well as interpreting the results. All authors have given their final approval of the version to be published.

## Pre-publication history

The pre-publication history for this paper can be accessed here:


